# State of the Art of Low‐Frequency Acoustic Modulation: Intensity Enhancement and Directional Control

**DOI:** 10.1002/advs.202410695

**Published:** 2025-04-17

**Authors:** Jingsong Xu, Yunfeng Ye, Tao Dong, Zhaochu Yang, Nuno Miguel Matos Pires, Yu Zhou, Fuyu Tao, Jin Wang, Junshan Zhang, Guoxi Luo, Libo Zhao, Qi Mao, Yangtao Wang, Zhuangde Jiang

**Affiliations:** ^1^ State Key Laboratory for Manufacturing Systems Engineering International Joint Laboratory for Micro/Nano Manufacturing and Measurement Technologies School of Instrument Science and Technology Xi'an Jiaotong University Xi'an 710049 China; ^2^ Chongqing Key Laboratory of Micro–Nano Systems and Intelligent Transduction Collaborative Innovation Center on Micro–Nano Transduction and Intelligent Eco‐Internet of Things National Research Base of Intelligent China; ^3^ China Electron Technology Group Corporation, Third Institute Beijing 100015 China; ^4^ Section of Radiation Medicine Teaching & Research the Fourth Military Medicine University Xi'an 710032 China; ^5^ College of Equipment Management and Support Engineering University of PAP Xi'an 710000 China

**Keywords:** acoustic metamaterials, directional control, high sound intensity, low‐frequency acoustic waves

## Abstract

High‐intensity low‐frequency acoustic sources with directivity play a significant role in various fields such as medical treatment, underwater communication, and environmental monitoring. However, the long wavelengths, strong penetration, and their tendency to easily diffract of low‐frequency acoustic waves make it challenging to achieve directional control and intensity enhancement. Thanks to the development of acoustic metamaterials, acoustic devices can now effectively manipulate low‐frequency acoustic waves at subwavelength scales with excellent acoustic performance. Currently, the directional control and intensity enhancement of low‐frequency acoustic waves mainly concentrate on source design and the modulation of propagation processes. These techniques employ acoustic resonance, focusing, and other phase control methods to achieve energy concentration and directional control of low‐frequency acoustic waves. Nevertheless, existing low‐frequency acoustic wave control techniques still face issues such as low energy efficiency, poor directional control, and limited controllable bandwidth. This paper systematically reviews methods for achieving high‐intensity emission and directional control of low‐frequency acoustic waves, comprehensively compares the advantages and disadvantages of various technologies, and discusses how to extend these methods to lower acoustic frequency bands, aiming to provide new insights for the development of miniaturized, efficient, and accurately directional ultra‐low frequency acoustic devices.

## Graphical abstract

High intensity low‐frequency sound sources hold significant value in many fields. However, their long wavelength, strong penetrability, and tendency to diffract make direction control and intensity enhancement challenging. Acoustic generators and metamaterial‐based acoustic devices still face issues such as low energy efficiency, poor directional control, and narrow bandwidth. Therefore, exploring miniaturized, efficient, and controllable acoustic devices is of great significance.

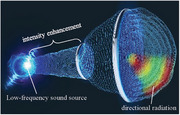

## 1 Introduction

The categorization of low‐frequency acoustic waves varies across different research fields, where the term “low frequency” is inherently relative and context‐dependent. In the scholarly discourse concerning the impact of low‐frequency acoustics on human physiology and psychology, the threshold is usually set at frequencies below 250 Hz.^[^ ^1^ ^]^ In the field of industrial acoustics, acoustic waves with frequencies below 1000 Hz are categorized as low‐frequency waves, which pose a significant threat to both the physical and psychological well‐being of humans, as well as to the precision and stability of sophisticated instruments and apparatuses.^[^ ^2^ ^]^ In the field of marine acoustics, particularly in shallow marine environments, the term “ultra‐low frequency sound” is designated for frequencies that are below the 100 Hz threshold,^[^ ^3^ ^]^ and infrasound (≤20 Hz) falls within the category of ultra‐low frequency sound.

The generation of low‐frequency acoustic waves is multifaceted, encompassing both natural^[^ ^4^, ^5^, ^6^ ^]^ and anthropogenic sources.^[^ ^7^, ^8^, ^9^ ^]^ The propagation characteristics of low‐frequency acoustic waves are influenced by their inherent low‐frequency and long‐wavelength, which reduce energy dissipation during propagation, enhance penetration capabilities, and facilitate long‐distance propagation. As the frequency decreases, these properties become more pronounced. However, it is these very properties that pose difficulties in energy focusing and directional emission of low‐frequency acoustic waves; especially in the natural environment, the low energy density of sound makes the localized enhancement of acoustic fields in the air particularly challenging. In addition, directional propagation technology for low‐frequency acoustic waves holds broad application prospects across multiple fields, including underwater communication,^[^ ^10^ ^]^ medical treatment,^[^ ^11^ ^]^ and industrial pipeline leak detection.^[^ ^12^ ^]^ Thus, research on the high‐intensity directional emission of low‐frequency acoustic waves, especially infrasound, is of great importance.^[^ ^13^ ^]^

Regarding the enhancement of sound intensity and directional control of low‐frequency acoustic waves, the primary technical approaches can be broadly categorized into two types: 1) Design the sound source: By structurally designing the acoustic generator or applying specific phase control to the sound source, the sound wave energy can be increased and focused to emit directional low‐frequency acoustic waves. 2) Modulation during propagation: This approach involves strategically placing specialized acoustic devices to modulate the wave propagation, thereby achieving directional control and energy focusing. Conventional acoustic manipulation equipment often corresponds to low energy efficiency and large size, resulting in a significant gap between theoretical research and practical application. Therefore, various new materials have been developed for the miniaturization of acoustic devices to enable their practical application, such as phononic crystals^[^ ^14^, ^15^ ^]^ and acoustic metamaterials (AMs).^[^ ^16^ ^]^ Both phononic crystals and acoustic metamaterials are artificially composite structural materials that possess acoustic properties not found in natural materials. Phononic crystals were proposed earlier, and some of their important properties, such as acoustic band structure and negative refraction, have stimulated basic and practical research on acoustic metamaterials.^[^ ^17^ ^]^ By designing the microstructure within metamaterials, it is possible to control the propagation characteristics of acoustic waves. As an emerging method for acoustic control in recent years, acoustic metamaterials hold a very promising future for applications.^[^ ^16^, ^18^, ^19^, ^20^ ^]^ They enable acoustic devices to manipulate acoustic waves at sub‐wavelength scales, offering significant potential for device miniaturization and further enhancing the practicality of acoustic device. It should be noted that in this article, the size of acoustic devices between *λ* and *λ*/10 (where *λ* is the wavelength of low‐frequency acoustic waves) is defined as sub‐wavelength scale, and the size smaller than *λ*/10 is defined as deep sub‐wavelength scale. Increasingly, sound intensity enhancement and directional control technologies are being developed on this foundation.

**Figure 1:**
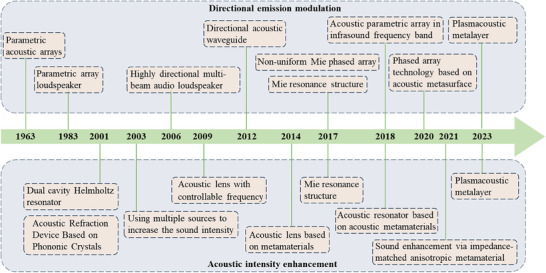
A timeline diagram of the development of low‐frequency sound enhancement technology and sound direction emission technology.

However, current research on the intensification of acoustic waves predominantly focuses on the ultrasonic range, with comparatively less attention given to the enhancement of low‐frequency sound. In existing research, low‐frequency acoustic waves are typically generated using electroacoustic transducers.^[^ ^21^, ^22^ ^]^ Nevertheless, their intensity often falls short of that produced by pneumatic generators.^[^ ^23^ ^]^ Unfortunately, pneumatic sound generators also face challenges such as high frequency and bulkiness. Further, in addition to designing different structures for generating acoustic waves, resonance chambers can be designed for sound sources to enhance the emitted sound intensity.^[^ ^24^, ^25^, ^26^ ^]^ With the support of acoustic metamaterials, acoustic resonators are also evolving toward high performance and miniaturization. However, acoustic resonances simultaneously raise the requirements for material stiffness and toughness. The emergence of plasmacoustic metalayers^[^ ^27^, ^28^ ^]^ can, to some extent, solve the dependency on materials, and they can achieve good control over acoustic waves within extremely small sizes. However, they also face issues such as complex external circuits and reliance on external high‐voltage power supplies. This technology, along with acoustic focusing based on acoustic lenses,^[^ ^29^, ^30^, ^31^ ^]^ belongs to the same category, which is achieved by modulating the phase of acoustic waves to focus and enhance the intensity of acoustic waves. Moreover, the significant diffraction effects exhibited by low‐frequency acoustic waves with long wavelengths also hinder their directional control. Currently, most research on directional control of acoustic waves focuses on high frequencies and ultrasonic ranges; while, low‐frequency acoustic wave directional emission technology is generally plagued by the bulkiness of acoustic devices and poor directional performance. Techniques such as parametric array technology^[^ ^32^ ^]^ and phased array technology^[^ ^33^ ^]^ have seen extensive application and reached a mature stage in ultrasonic focusing. Their application in low‐frequency acoustic focusing is relatively limited, primarily due to the complexity and the associated high costs. However, these techniques offer the best directional effects compared to other methods, such as acoustic lenses^[^ ^34^ ^]^ and acoustic metamaterials.^[^ ^35^ ^]^ Inspired by the principles of optical lenses, acoustic lenses are also used to manipulate acoustic waves, primarily for focusing ultrasound. Acoustic lenses can be made from a variety of materials, including plastics, metals, and composites. The choice of material depends on the desired acoustic properties, such as impedance matching, absorption, and dispersion. Compared to the aforementioned techniques that manipulate the phase of acoustic waves to enhance directionality, Mie resonance structures^[^ ^36^, ^37^, ^38^ ^]^ can scatter energy in the desired direction, thereby reducing energy loss during acoustic wave manipulation. However, their complex structural design and requirements for material stiffness and other properties also limit their application in certain areas. Plasmacoustic metalayers^[^ ^28^ ^]^ can achieve directional control at deep sub‐wavelength scales to some extent through the coherence of reflected waves and original waves; however, their manipulation of ultra‐low frequency acoustic waves has not yet been studied. The development of low‐frequency acoustic wave intensity enhancement and directional emission modulation over time is shown in **Figure** **1**.

Differing from directional and focusing technologies for ultrasonic waves, research on intensity enhancement and directional emission of low‐frequency acoustic waves remains incomplete; especially, the directional emission modulation of infrasound is unexplored according to the available literature. Therefore, this article aims to provide a comprehensive and systematic review of the enhancement and directional technologies for low‐frequency acoustic waves, identify the challenges faced by current methods, and look forward to potential future development directions. Through this review, we aim to provide insights into technologies for enhancing the intensity and directional control of low‐frequency sound.

## 2 Current Status of Low‐Frequency Acoustic Waves Application

Directional acoustic emission technology has shown significant importance in various applications, including focused acoustic waves for medical purposes, target localization in sonar systems, and directional emission and reception in acoustic communications.^[^ ^10^, ^39^ ^]^ However, current research almost exclusively focuses on mid‐to‐high frequency acoustic waves; owing to the inherent difficulty in focusing low‐frequency acoustic waves, particularly infrasound, the application for low‐frequency acoustic waves is significantly constrained. Meanwhile, there is a significant demand for high‐intensity low‐frequency acoustic waves. Low‐frequency acoustic waves empowered by acoustic energy enhancement techniques have significant application value in acoustic biological effect research, bird repelling at airports, and boiler soot removal, among other areas.^[^ ^40^ ^]^ Moreover, an increasing number of applications require achieving high sound pressure levels (SPL) in the far field with low‐frequency acoustic waves such as non‐lethal acoustic weapons^[^ ^41^ ^]^ and techniques involving the acoustic dispersion of fog,^[^ ^42^ ^]^ among others.

Underwater acoustic communication technology is a core technology currently applied in ocean resource exploration, wide‐area ocean data collection, and distributed ocean environment detection.^[^ ^43^ ^]^ The use of low‐frequency acoustic waves in underwater communication has existed for a long time; the characteristics of long wavelength and slow attenuation make it possible to communicate over long distances.^[^ ^44^ ^]^ The underwater acoustic communication based on directional acoustic emission technology has extraordinary application value, and it is of great significance to many fields such as ocean exploration.^[^ ^45^ ^]^ First of all, directional acoustic emission technology can concentrate wave energy, thereby extending detection distances.^[^ ^46^ ^]^ Second, underwater communication with directional transmission can increase the detectability of the signal. Especially in the underwater inhomogeneous environment, the accuracy and stability of underwater communication will be greatly improved.^[^ ^47^ ^]^

The low‐frequency acoustic wave also plays an important role in various aspects of life. For example, in environmental monitoring, infrasound is closely associated with factors such as wind and temperature distribution in the atmosphere. By analyzing the propagation characteristics of infrasound waves in various atmospheric activities, such as sandstorms, tornadoes, and atmospheric electromagnetic wave disturbances, the properties and patterns of infrasound in certain natural phenomena can be discovered, thereby enabling the monitoring of these activities through infrasound waves. Wei et al.^[^ ^48^ ^]^ proposed a novel acoustic interference technology using strong low‐frequency acoustic waves. They studied the interference of strong low‐frequency acoustic waves on cloud layers and precipitation, proposing that the triggering and periodic effects of acoustic waves on cloud layers are key responses to acoustic atmospheric interference, which is of great significance for further research on atmospheric interference technology based on low‐frequency strong acoustic waves. Based on the jet blocking principle, they used a modulation frequency of 50 Hz to modulate the airflow to emit acoustic waves, with a combined SPL of up to 136.7 dB at low frequencies.

In the medical field, the application of low‐frequency acoustic waves has been extensively studied and practiced. Regarding physiological mechanisms, low‐frequency acoustic waves can improve blood circulation by stimulating cells.^[^ ^49^ ^]^ In addition, low‐frequency acoustic wave therapy has also shown potential benefits for the respiratory system, such as enhancing the overall function of the body by increasing the functional reserves of the respiratory system.^[^ ^50^ ^]^ Low‐frequency acoustic waves can also safely stimulate bone tissue, promoting the maintenance and regeneration of bone tissue.^[^ ^51^ ^]^ In addition, infrasound, with its lower frequency, possesses an even more powerful and direct effect; specific frequencies of infrasound can resonate with human organs, affecting the human body. In the healthcare sector, infrasound is used to develop diagnostic instruments to check whether human organs are functioning normally. For example, infrasound can directly alter the ultrastructure of tumor cells and may make several types of cancer sensitive to chemotherapy, and it can cause changes in membrane permeability and metabolism of radiation cells.^[^ ^11^ ^]^ Based on the effects of infrasound on the human body, high‐intensity infrasound also plays a significant role in public safety, being crucial for riot control^[^ ^52^, ^53^ ^]^ and maintaining social peace and stability. The specific effects can be achieved through two aspects. On the one hand, the physical effects of infrasound directly impact the body's condition, including causing nausea and vomiting. On the other hand, by influencing the auditory system, infrasound indirectly affects the brain's cognitive functions, such as distraction and memory decline.^[^ ^49^ ^]^

What's more, the strong penetrating power of infrasound gives it potential value in industrial applications, such as for detecting the internal structure of materials or for non‐destructive testing. For example, in pipeline leak detection, the acoustic signals generated by leaking gas propagate along the pipeline. The high‐frequency components of the acoustic wave attenuate rapidly; while, the low‐frequency components can travel long distances. Therefore, detecting pipeline leaks through acoustic methods is superior to conventional methods, offering higher sensitivity, longer detection distances, lower false alarm rates, faster leak detection, and stronger real‐time capabilities.^[^ ^12^ ^]^ Moreover, Seiffert et al. used high‐intensity, low‐frequency sound to remove powder layers, overcoming the adhesion and cohesion forces formed when powder electrostatically deposited onto metal surfaces by controlling the SPL and frequency.^[^ ^54^ ^]^ Meanwhile, low‐frequency acoustic waves also play a role in noise control,^[^ ^55^ ^]^ fire extinguishing,^[^ ^56^ ^]^ and other fields.

To summarize, due to their strong penetrating power and long transmission distances, low‐frequency acoustic waves have already played a significant role in various aspects of our lives, facilitated by directional acoustic emission and acoustic energy enhancement technologies. This paper will systematically review the current research status of acoustic energy enhancement and directional emission technologies for low‐frequency acoustic waves. It is hoped that by summarizing existing studies, more practical acoustic manipulation methods and devices can be improved and explored.

## 3 Intensity Enhancement of Low‐Frequency Acoustic Waves

### 3.1 Difficulties and Challenges of High‐Intensity Low‐Frequency Acoustic Wave Generation

As outlined in Section 2, high‐intensity low‐frequency acoustic sources hold significant application value across numerous fields. Although many high‐performance speakers have been used in production and daily life, they primarily generate acoustic waves in the mid‐to‐high frequency range. High‐intensity low‐frequency sound sources are rare in open spaces due to the challenges involved in generating such acoustic waves. While speakers can increase acoustic radiation efficiency by raising input electrical power, low‐frequency acoustic radiation requires large‐sized diaphragms and significant displacement reciprocation, which is difficult to achieve in engineering.^[^ ^24^ ^]^ In addition, in applications requiring high‐power acoustic wave (high intensity) output, electroacoustic generators may be less effective than other types of sound generators, such as pneumatic generators. Aerodynamic acoustic sources possess high radiation efficiency and intensity, commonly used for creating high‐intensity sound sources.^[^ ^23^ ^]^ Aerodynamic acoustic sources typically consist of airflow modulators and horns, capable of generating sound power up to ten thousand watts. In addition, aerodynamic sound sources can also generate jet sound using high‐pressure air.^[^ ^57^ ^]^ The sound intensity generated by these sources can be very high, but they are mainly composed of high‐frequency components. Enhancing the intensity of low‐frequency components is challenging.

**Figure 2:**
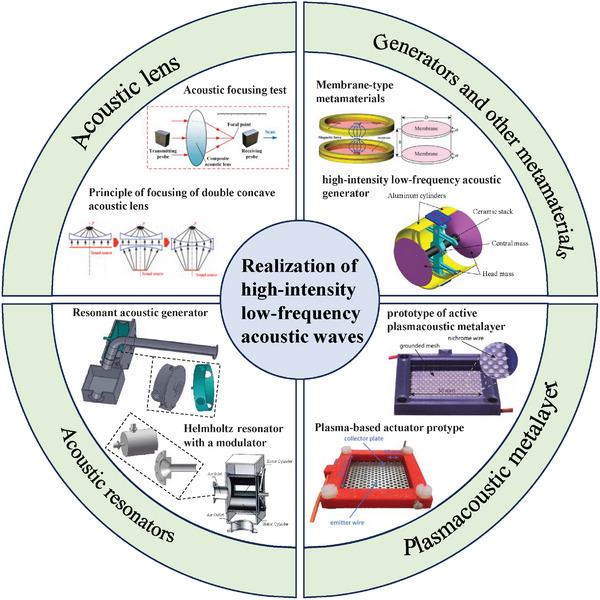
Various high‐intensity low‐frequency acoustic wave realization technologies. 1) Acoustic lens. Reproduced with permission.^[^ ^58^ ^]^ Copyright 2020, Elsevier. 2) Acoustic resonators. Reproduced with permission.^[^ ^13^, ^24^ ^]^ Copyright 2011, SAGE Publications Inc. and Copyright 2024, PLOS. 3) Plasmacoustic metalayer. Reproduced with permission.^[^ ^27^, ^28^ ^]^ Copyright 2023, Sergeev et al. and Copyright 2023, Springer Nature. 4) Generators and other metamaterials. Reproduced with permission.^[^ ^21^ ^]^ Copyright 2013, ASA.

The methods for generating high‐intensity low‐frequency acoustic waves have been continuously explored, with typical approaches focusing on improving the structure of the sound source and optimizing the process of sound propagation. On the one hand, the strategic design of specialized structures for acoustic generators, particularly acoustic resonators, enables the direct generation of high‐intensity, low‐frequency acoustic waves. On the other hand, external devices are used to adjust the phase of acoustic wave propagation, such as acoustic lenses capable of focusing acoustic waves. By coherently superimposing the modulated wave with the original wave to enhance the sound intensity, the shortcomings of insufficient sound intensity from the sound generator can be compensated. To match high‐intensity sound sources, these devices typically have dimensions comparable to the wavelength, which is disadvantageous for generating low‐frequency acoustic waves. With the development of new materials, these issues are expected to be resolved. This section will focus on several commonly used techniques capable of generating high‐intensity low‐frequency acoustic waves, as illustrated in **Figure** **2**, aiming to provide a comprehensive perspective on the generation of high‐intensity low‐frequency acoustic waves.

### 3.2 High‐Intensity Low‐Frequency Sound Generation

The generation of high‐intensity low‐frequency acoustic waves relies on large‐scale sound source systems and the support of external auxiliary devices. With the application of new materials such as acoustic metamaterials, both sound generation equipment and devices for controlling acoustic wave propagation are evolving toward smaller size and higher performance. Figure 2 lists some common techniques for generating high‐intensity low‐frequency sound, among which acoustic metamaterials cover a wide range. This review summarizes some classic methods for generating and manipulating low‐frequency acoustic waves based on acoustic metamaterials, including acoustic lenses, acoustic resonators, and plasmacoustic metalayers, some of which leverage the principles of acoustic metamaterials. In addition, other sound enhancement technologies utilizing acoustic metamaterials, as well as sound generators, are presented to provide readers with a comprehensive understanding of the current research landscape in low‐frequency acoustic intensity enhancement.

#### 3.2.1 Acoustic Lens Focus

Common methods to achieve acoustic focusing include medium reflection focusing, phased array focusing, and acoustic lens focusing.^[^ ^59^ ^]^ Acoustic lens focusing is a low‐cost, high‐efficiency method in practical applications, especially in underwater systems,^[^ ^58^, ^60^ ^]^ as shown in Figure 2. Acoustic lenses are utilized across various fields, from ultrasonic imaging to non‐destructive testing. Compact and efficient acoustic lenses are crucial for achieving the miniaturization and integration of acoustic devices. However, there are relatively few reports on using acoustic lenses to achieve low‐frequency acoustic focusing to enhance sound intensity, and most studies focus on ultrasonic focusing.^[^ ^30^, ^31^ ^]^ Although this contributes to the wide application of acoustic lenses in biomedical and industrial fields, it leaves a gap in the exploration of low‐frequency applications.

**Figure 3:**
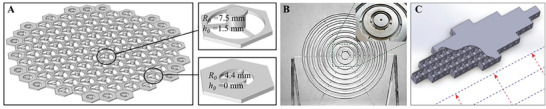
Different types of acoustic lenses. A) Resonant‐type structural Luneburg lens with 109 units. Reproduced with permission.^[^ ^67^ ^]^ Copyright 2024, Zhao et al. B) The schematic diagram of the 3D axisymmetric acoustic gradient index lens. Reproduced with permission.^[^ ^61^ ^]^ Copyright 2014, Springer Nature. C) Acoustic metamaterial lens consisting of 2D FCOC unit cells. Reproduced with permission.^[^ ^77^ ^]^ Copyright 2015, AIP Publishing.

Various metamaterial‐based acoustic lenses have been employed to enhance the focusing of low‐frequency acoustic waves, such as planar lenses,^[^ ^61^ ^]^ gradient‐index lenses,^[^ ^62^ ^]^ Fresnel acoustic lenses,^[^ ^63^, ^64^ ^]^ and tunable acoustic lenses,^[^ ^65^ ^]^ among others. The acoustic frequencies reported in these studies typically fall within the range of several kilohertz and above. Liu et al.^[^ ^66^ ^]^ reported a novel high‐efficiency acoustic focusing lens based on a Helmholtz resonator that can achieve deep subwavelength resolution for acoustic focusing; while, the applicable frequency range of this lens is from 1 to 4 kHz. The proposed Helmholtz resonator structure has a thickness of only *λ*/15 (*λ* is the wavelength of acoustic waves), which can be further reduced to *λ*/30 by sacrificing some acoustic reflection, much smaller than the *λ*/8 of coiled structures. However, due to this pursuit of device miniaturization, a significant loss of concentrated energy occurs. Zhao et al. proposed a new type of Luneburg lens structure with local resonators that can achieve subwavelength focusing within a low‐frequency range, and the lens exhibited good performance within the 2 kHz‐4 kHz.^[^ ^67^ ^]^ In addition, the Luneburg lens plays a significant role in the section on gradient lenses. Fu et al.^[^ ^68^ ^]^ used an acoustic inverse reflector based on a mirrored Luneburg lens to add a reflection boundary at the focus of the Luneburg lens; so that, the incident acoustic waves converged to the focus and then reflected, thereby achieving the concentration and enhancement of acoustic energy. Similarly, Xie et al.^[^ ^69^ ^]^ used a Luneburg lens based on metamaterials through which the incident sound waves converged to the focus and then reflected back. The unique gradient refractive index distribution directly bended the incident plane wave and focused it to the focus on the spherical surface opposite the lens, realizing the concentration of sonic energy in a specific area. Zhao et al.^[^ ^67^ ^]^ proposed a novel structural Luneburg lens with a local resonator capable of focusing acoustic waves at subwavelength scales. As shown in **Figure** **3**A, the lens configuration comprised 109 units, which offered broadband focusing at low frequencies by adjusting the geometric parameters. However, these acoustic Luneburg lenses suffered from narrow bandwidth and challenges in integration. Coiled space structures had also been used for the design of gradient acoustic lenses.^[^ ^70^ ^]^ Yuan et al. utilized a gradient acoustic metasurface with a space‐coiling structure to achieve efficient conversion from cylindrical to plane waves and plane wave focusing within a deep subwavelength thickness range (<*λ*/10) through a single‐layer metasurface.^[^ ^71^ ^]^ Moreover, using gradient acoustic lenses, acoustic radiation patterns could be manipulated, such as focusing, tunable transmission,^[^ ^72^ ^]^ and reflection.^[^ ^73^ ^]^ For example, Peng et al.^[^ ^74^ ^]^ designed a flat subwavelength lens that could focus acoustic waves through an acoustic grating with a gradient effective refractive index. They studied acoustic gratings with curled slits and found that these gratings can act as tunable impedance and refractive index materials for acoustic waves. The effective parameters depend on the geometry of the slits and are independent of frequency, providing the possibility for focusing acoustic waves over a broad bandwidth. Further, the application scope of acoustic lenses is extensive. Jahdali proposed two types of impedance‐matched acoustic lenses customized by acoustic metasurfaces for focusing acoustic waves in water and air, respectively. The focusing effects of both lenses were demonstrated through finite element simulations, indicating that high energy is transmitted through the lenses over a wide frequency range due to matched impedance and an intrinsic non‐resonant mechanism.^[^ ^75^ ^]^ In addition, acoustic lenses based on phononic crystals have been designed and manufactured to focus incident acoustic waves.^[^ ^18^, ^76^ ^]^ Besides, there are some reports on acoustic lens focusing within the frequency range of several hundred hertz. For example, Li et al.^[^ ^61^ ^]^ designed and implemented ultrathin planar acoustic lenses capable of guiding acoustic waves to converge in 3D space, as shown in Figure 3B. These lenses can enhance acoustic energy by 15 dB at the focal point at an acoustic wave frequency of 815 Hz, with very high transmission efficiency, and the lens thickness is only ≈1/6 of the working wavelength. As shown in Figure 3C, Park et al.^[^ ^77^ ^]^ designed a 2D acoustic metamaterial convex lens composed of multiple face‐centered‐orifice‐cubic (FCOC) unit cells, which could achieve good focusing effects at an acoustic wave frequency of 450 Hz.

Further, several studies have explored the use of acoustic metamaterials for acoustic wave focusing to achieve sound intensity enhancement. For instance, Toulis^[^ ^78^ ^]^ proposed a spherical structure for acoustic focusing in 1963, which achieved high‐precision acoustic wave focusing and significantly enhanced directivity gain. Guenneau et al.^[^ ^79^ ^]^ designed a double “C” resonator that exhibited negative refraction effects at low‐frequency acoustic waves, successfully realizing acoustic wave focusing. Gong et al.^[^ ^80^ ^]^ developed a gradient‐tunable acoustic metasurface based on Helmholtz resonators, which could significantly enhance sound intensity at the focal point on a deep subwavelength scale. However, these methods for acoustic focusing generally suffer from a narrow operable frequency band and are susceptible to environmental interference, which limits their widespread practical application.

In summary, a variety of special structures and materials (especially metamaterials) has been used to design and manufacture acoustic lenses and achieve the focusing of acoustic waves at deep subwavelengths. Extending the focusing performance of acoustic lenses to very low frequencies, particularly infrasound, necessitates the use of larger radii of curvature or sophisticated structural configurations.

#### 3.2.2 Acoustic Resonators

In research related to high‐intensity low‐frequency acoustic generators, the use of resonators can enhance the intensity of low‐frequency acoustic in enclosed spaces, as shown in Figure 2.^[^ ^13^, ^24^ ^]^ The Helmholtz resonator, due to its relatively simple structure, is the most commonly used type of resonator. Typically, a Helmholtz resonator consists of a large enclosed volume and an extended neck with a small opening. Within a Helmholtz resonator, acoustic resonance can be generated at specific frequencies; thus, enabling the enhancement of sound intensity at tunable frequencies.

**Figure 4:**
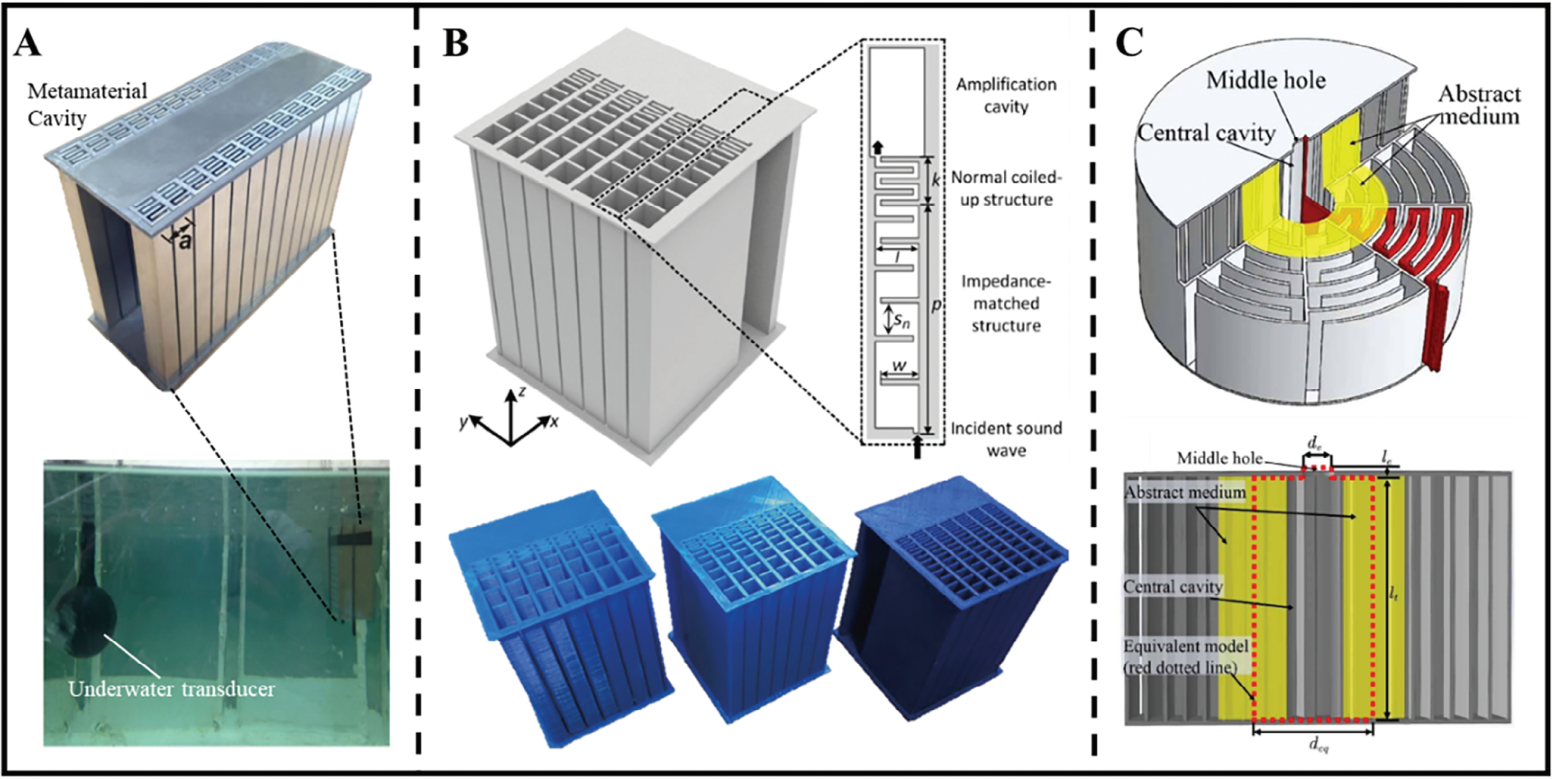
Different types of acoustic resonators. A) Acoustic metamaterial cavity composed of double coiled‐up space‐like structures (left) and schematic and photograph of the experimental apparatus designed to measure SPL in an underwater environment (right). Reproduced with permission.^[^ ^83^ ^]^ Copyright 2014, Springer Nature. B) Schematic of acoustic meta‐structure resonators and 3D‐printed meta‐structure resonators with various structural parameters. Reproduced with permission.^[^ ^84^ ^]^ Copyright 2018, IOP Publishing. C) Schematic diagram and cross‐sectional view of the internal structure of the Mie‐Helmholtz resonator. Reproduced with permission.^[^ ^37^ ^]^ Copyright 2021, IOP Publishing.

Impedance matching is crucial for generating high‐intensity acoustic waves when using resonators to enhance acoustic intensity. Zhang et al., based on fluid dynamics, aeroacoustics, and electroacoustic analogy principles, investigated the impedance calculation methods for modulators and dual‐chamber Helmholtz resonators. They provided a design scheme for impedance matching in a two‐chamber Helmholtz low‐frequency high‐intensity acoustic generator. This method has a guiding significance for the design of high‐intensity acoustic generators in enclosed spaces.^[^ ^81^ ^]^ Similarly, Song et al. developed a highly miniaturized double‐walled metamaterial cavity that successfully enhanced the emission of low‐frequency sound. The device used a Fabry–Perot resonance mode for sound enhancement, determined by the quality factor and effective cavity volume, with a 10 dB sound emission enhancement near 1060 Hz.^[^ ^82^ ^]^ In another study by Song et al.,^[^ ^83^ ^]^ they also developed an acoustic metamaterial cavity composed of a double‐coiled spatial structure, which could provide up to 13 dB of SPL gain within the scale of 1/10 of the incident acoustic wavelength, as shown in **Figure** **4**A. The amplification performance of the cavity was also evaluated in an underwater environment, demonstrating a significant acoustic signal enhancement of 3–4 dB within the frequency range of 3–25 kHz. Although the involved acoustic waves operate at relatively high frequencies, this study presents a novel approach for enhancing underwater acoustic propagation. Further, research on sound enhancement has gradually expanded to lower frequencies, including even infrasound. As illustrated in Figure 4B, Jia et al.^[^ ^84^ ^]^ designed a novel acoustic resonator unit based on acoustic metamaterials to achieve amplification of acoustic energy. At a resonant frequency of 625 Hz, the unit achieved a maximum SPL gain of 17.2 dB. Landi et al.^[^ ^85^ ^]^ utilized the Acoustic Purcell Effect to design a sub‐wavelength cavity structure that could enhance the emission of sound sources over the full frequency range, especially at the resonance frequency of ≈485 Hz, where the sound power increased by 23 dB. Zhang et al. developed a low‐frequency acoustic generator with a resonant cavity for enhancing acoustic intensity in open spaces, as shown in Figure 2. This aerodynamic acoustic generator is used to radiate high‐intensity acoustic waves at 52 Hz. The sound intensity at 52 Hz is 124 dB at a distance of 1 meter. By combining the Helmholtz resonator with an airflow modulator, the airflow resonance in the resonator enhances the air pressure pulsation within the chamber and increases the disturbance of acoustic radiation to the air. This improves the sound intensity and radiation efficiency in the low‐frequency range. Boesch and Reiff^[^ ^26^ ^]^ also used an airflow modulator and a Helmholtz resonator to construct a high‐intensity infrasonic wave generator with an acoustic intensity greater than 140 dB. Subsequently, they built a dual‐chamber resonator system, achieving an acoustic intensity above 155 dB. As shown in Figure 2, Dong et al.^[^ ^86^ ^]^ also studied the working mechanism of an infrasonic wave generator composed of a modulator and a Helmholtz resonator: by combining the Helmholtz resonator with an airflow modulator, the airflow resonance in the resonator enhances the air pressure pulsation within the chamber, increasing the disturbance of acoustic radiation to the air. This improves the sound intensity and radiation efficiency in the low‐frequency range; they also manufactured a prototype device with an infrasonic pressure level exceeding 162 dB below 3 Hz. These examples all generate high‐intensity acoustic waves at lower frequencies; thus, exhibiting excellent acoustic performance.

In addition, Yuan et al. proposed an efficient acoustic energy harvester suitable for the low‐frequency band, with the proposed Helmholtz resonator designed for acoustic pressure amplification.^[^ ^25^ ^]^ Zhang et al. utilized Mie resonance structures to design tunable acoustic antennas, achieving a significant enhancement in radiated power sound intensity.^[^ ^87^ ^]^ However, their ability to enhance sound intensity is limited. As shown in Figure 4C. a Mie–Helmholtz structure (M–H structure) based on Mie resonance was designed by Zeng et al., which could further enhance far‐field SPL and unidirectional directivity by utilizing the high acoustic pressure inside the central cavity.^[^ ^37^ ^]^ The drawback of this structure lies in its complex design. Zhao et al.^[^ ^88^ ^]^ proposed a sub‐wavelength anisotropic acoustic metamaterial structure to enhance the radiation of monochromatic multipolar sound sources. The structure realizes the localization and enhancement of the sound field near the Mie resonance frequency by designing a metamaterial with a specific radial speed of sound and infinite tangential density, significantly improving the radiation efficiency of the sound source.

To extend the wave manipulation capabilities of the aforementioned acoustic resonators to very low frequencies and even infrasound frequencies, on the one hand, optimizing the acoustic impedance matching can reduce reflections; and thus, improve energy utilization efficiency. On the other hand, the size and resonant structure of the resonator should be adjusted to ensure that the resonant frequency matches the very low‐frequency acoustic waves. If the size of the acoustic resonator can be controlled to an appropriate size, it will exhibit outstanding performance in enhancing sound intensity at very low frequencies (especially in the infrasound range), thereby offering broad application prospects.

#### 3.2.3 Plasmacoustic Metalayer

Plasma‐based acoustic metalayers differ fundamentally from traditional transducers. The sound field is directly controlled through the interaction of air particles with the ionized air layer generated in the metamaterial. This transduction principle allows for a faster response to input acoustic signals compared to membrane‐based interfaces and significantly increases the control bandwidth. Plasma‐based acoustic metalayers offer a promising concept for the study of acoustic metamaterials as the phase of acoustic waves can be controlled in a broadband manner.^[^ ^27^ ^]^

Passive components for manipulating acoustic waves typically correspond to larger volumes (positively correlated with wavelength) and narrower operating bandwidths.^[^ ^89^ ^]^ However, devices based on narrowband resonances, such as locally resonant metamaterials, can still function in this state but at the cost of sacrificing a significant amount of bandwidth, thereby offering notable but limited possibilities for acoustic wave bending and focusing. Active control schemes can avoid this issue. Applied to subwavelength acoustic resonators, active methods have led to solutions with higher reconfigurability and bandwidth. However, even in active schemes, such as those based on electric or piezoelectric transducers, the achievable bandwidth cannot be arbitrarily extended. Sergeev et al. utilized the inherent non‐inertial dynamics of ultrathin air plasma layers to construct a fundamentally broadband active plasma layer, as shown in Figure 2. This allows for a significant reduction in the size of acoustic devices, even down to *λ*/1000. The ionization of the air medium can be achieved through the design of corona discharges; and by adjusting the acoustic impedance of the surrounding medium, it is possible to tune the amplitude and phase of the reflected acoustic waves. When the phase of the reflected wave is adjusted to match that of the incident wave, the amplitudes of the acoustic waves can be superimposed, thereby obtaining high‐intensity acoustic waves. Due to the ability to actively manipulate plasma‐based acoustic metalayers with very small characteristic sizes, the dimensions of the structures are satisfactory even at low frequencies or in the infrasonic band.

However, a current limitation of plasma‐based sound intensity enhancement technology is that the maximum incident sound pressure level controllable by the device is relatively low. It depends on the target impedance, and for the prototype shown in Figure 2, it is typically within the range of 85–100 dB. Reproduced with permission.^[^ ^27^ ^]^ Copyright 2023, Sergeev et al. Higher SPL requires higher driving voltage levels; however, this may lead to exceeding the dynamic range of corona discharge voltages.

#### 3.2.4 Other Methods for Sound Intensity Enhancement

In addition to the three methods mentioned above to enhance sound intensity, there are other ways to generate high‐intensity low‐frequency acoustic waves.

##### Acoustic Metamaterials

Beyond the previously discussed acoustic metamaterials, including acoustic lenses, acoustic resonators, and plasma‐inspired acoustic metasurfaces, numerous other types of acoustic metamaterials have been explored and engineered to significantly boost acoustic intensity at low frequencies. Based on their structural characteristics and their response properties to acoustic waves, acoustic metamaterials are classified into the following categories: locally resonant structures, Helmholtz resonator structures, membrane‐type structures, coiled space structures, and other structures of acoustic metamaterials, as shown in Figure 2. However, locally resonant acoustic metamaterials (LRAM) have inherent limitations. Typically, LRAM is limited to manipulating acoustic waves within a narrow bandwidth near the resonant frequency range. Unlike LRAM, AMs designed with spatial coiling mechanisms can manipulate acoustic waves over broadband and are smaller in size. The spatial coiling structure achieves this by extending the propagation path of acoustic waves to delay the transmission phase, thereby constructing a fluid medium with an extremely slow equivalent velocity.^[^ ^70^, ^90^ ^]^ Zigzag channels are a typical way of path extension, and it has been demonstrated that space‐coiled AMs with zigzag channels can achieve multiple resonance modes within the low‐frequency range, resulting in unusual acoustic parameters. Sound enhancement through AMs is generally achieved by changing the propagation characteristics of acoustic waves; so that, acoustic waves are localized inside the metamaterial structure. This localization causes the amplitude of acoustic waves inside the structure to increase significantly, forming a strong acoustic field enhancement effect.

**Figure 5:**
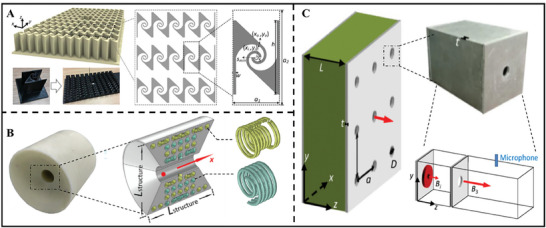
A) Schematic diagram of anisotropic metamaterial array and enlarged view of the metamaterial unit. Reproduced with permission.^[^ ^92^ ^]^ Copyright 2021, Elsevier. B) A compact acoustic device that combines a spiral coil and an acoustic resonance structure enables sound intensity enhancement and directional sound beaming. Reproduced with permission.^[^ ^94^ ^]^ Copyright 2018, APS. C) A high‐Q acoustic metasurface capable of enhancing planar acoustic waves and generating collimated acoustic beams. Reproduced with permission.^[^ ^95^ ^]^ Copyright 2019, APS.

Other acoustic devices constructed based on acoustic metamaterials have also demonstrated excellent capabilities in manipulating acoustic waves. For example, Wen et al.^[^ ^91^ ^]^ proposed an acoustic topological waveguide (ATW) based on the quantum valley Hall effect. By designing phonon crystal materials with different topological properties and using topology optimization methods to adjust their structural parameters, the localization and enhancement of acoustic waves at the waveguide interface are realized. Jia et al.^[^ ^92^ ^]^ used 3D printing technology to manufacture a bidirectional spiral‐shaped metamaterial and its 2D array, achieving a sound energy enhancement of over 10.75 dB within the spiral‐shaped unit in the frequency range of 4.8–5.8 kHz, as shown in **Figure** **5**A. However, these acoustic metamaterials mentioned above are made of hard materials, and their performance is not easily adjustable. In contrast, membrane‐type AMs are easier to achieve tunable acoustic properties.^[^ ^93^ ^]^ Nevertheless, membrane‐type AMs have the characteristics of low structural stiffness, poor load‐bearing capacity, and rapid aging, and the acoustic performance of the structure is unstable, with any small changes potentially causing a shift in the operating frequency. Overall, these acoustic metamaterials provide a solid foundation for the miniaturization of acoustic control devices and the flexible manipulation of low‐frequency acoustic waves, marking a major direction for the future development of acoustic equipment.

##### Acoustic Generators

Lan et al. designed a new electromagnetic high‐power underwater ultra‐low frequency sound source by establishing a theoretical model of electromagnetic, magnetic–mechanical, and force‐vibration conversion. The sound source reaches 186 dB at 73 Hz, with excellent ultra‐low frequency response and stability, providing an efficient, miniaturized, and lightweight sound source solution for marine environment exploration.^[^ ^22^ ^]^ Scarpitta et al. developed an electroacoustic transducer that emits very low‐frequency and high‐power acoustic waves underwater. The transducer consists of multiple electroacoustic motors and a flexible enclosure to which the electroacoustic motors are connected to the housing by an assembly device and transmit vibrations.^[^ ^96^ ^]^ The flexible housing is designed to enhance vibration amplitude in the very low‐frequency range; however, the maximum sound pressure level of this method is limited. Lubnow et al. developed a low‐frequency sound source that produces high‐intensity, low‐frequency sound by burning an explosive mixture in an explosion chamber at its bottom that is open to water.^[^ ^97^ ^]^ Moreover, Mosca et al. designed a Janus–Hammer Bell (JHB) transducer with high electro–acoustic efficiency (>60%) and compactness. The transducer worked on the 450–550 Hz bandwidth and reached a source level above 200 dB (ref. 1 µPa at 1 m) with 1 kW excitation and full immersion capability, as shown in Figure 2. These methods focus on enhancing sound intensity directly at the source of generation, without relying on external devices, significantly reducing the complexity of the overall equipment.

In addition, in some studies, both enhancement and directional radiation of acoustic waves have been achieved simultaneously. For example, Fan et al. proposed a cylindrical acoustic device that combines a spiral coil structure and acoustic resonance structure, achieving sound intensity enhancement and directional emission effects of a monopole low‐frequency sound source at the deep subwavelength scale,^[^ ^94^ ^]^ as shown in Figure 5B. Song et al.^[^ ^95^ ^]^ proposed a high‐Q acoustic metasurface that forms plane sound waves in a constructed Fabry–Perot cavity and obtains high‐intensity collimated sound beams, as shown in Figure 5C. Although some acoustic focusing methods also involve the orientation of acoustic waves, these methods often focus acoustic waves on a point without achieving high‐energy radiation in a certain direction. Therefore, in this paper, acoustic wave focusing and directional emission are distinguished.

### 3.3 Comparison of Various High‐Intensity Sound Generation Methods

As mentioned above, there are typically two ways to achieve high sound intensity at low frequencies: improving the structural design of the sound source and using external sound intensity enhancement devices. Both approaches rely on the use of acoustic metamaterials for device miniaturization. The commonly used methods for generating low‐frequency, high‐intensity acoustic waves have been extensively reviewed in the third section. Here, **Table** **1** lists the advantages and disadvantages of different methods, aiming to explore the future development direction for achieving high‐intensity low‐frequency acoustic waves.

**Table 1:** Comparison of different high‐intensity acoustic wave generation technologies.

Technical solution	Advantages	Disadvantages	Acoustic intensity/acoustic power	Effective working bandwidth/frequency	Refs.
Acoustic lens focus	• Energy concentration • High precision • High flexibility	• Limited focus range • Narrow bandwidth	SPL gain: 15 dB at 815 Hz^[^ ^61^ ^]^	500–1100 Hz^[^ ^61^ ^]^	[66]
Acoustic resonators	• Low energy loss • Compact structure • Low cost	• Frequency‐dependent • Larger size at low frequencies	161 dB at 3 Hz and higher‐intensity infrasound below 3 Hz can be generated^[^ ^13^ ^]^	≤11 Hz^[^ ^13^ ^]^	[24, 26, 98]
Plasmacoustic metalayer	• Low power • Wide bandwidth • Deep subwavelength scale	• High sound intensity corresponds to high‐power excitation	Metalayer prototype consumes electrical power of 2.5 W to maintain the ionization at 8 kV bias voltage^[^ ^28^ ^]^	20–2000 Hz^[^ ^28^ ^]^	[27, 28]
Acoustic generators and other metamaterials	• Small size • High intensity • High efficiency • Wide application	• Complex processing • High cost • Poor normalization	200 dB (ref. [1] µPa at 1 m)^[^ ^21^ ^]^	450–550 Hz^[^ ^21^ ^]^	[22, 92, 94, 95, 96]

As shown in Table 1, acoustic metamaterials have laid the foundation for the research of other acoustic devices. However, their complex structural designs and the wide variety of acoustic structures have also led to some deficiencies in device performance. Acoustic lenses are passive devices that assist in enhancing sound intensity. They can flexibly control the focal position of acoustic waves, but they also have limitations in terms of the distance of acoustic focusing and the bandwidth of sound they can manipulate. Acoustic resonators are common structures for enhancing sound intensity. They have a high energy utilization efficiency, are low‐cost, and offer design flexibility. However, acoustic resonators are typically only effective at specific frequencies or within a narrow frequency range, which limits their use in broadband applications. Benefiting from the advancements in acoustic metamaterials, acoustic resonators can now achieve low‐frequency acoustic wave intensity enhancement at smaller scales. However, this also necessitates complex computational and experimental validation for designing efficient acoustic resonators, potentially increasing development time and costs. The application of plasmacoustic metalayers offers broad prospects for acoustic wave manipulation at deep subwavelength scales. Their active control method can achieve broadband acoustic wave manipulation, but it also introduces complex circuit structural designs, and the manipulation of acoustic waves at low frequencies has not yet been fully validated. Further, the table also presents the optimal sound intensity gains and other performances introduced in the latest current research for various sound intensity enhancement methods, along with their corresponding effective operational bandwidth/frequency. Among these, acoustic resonators and plasmacoustic metalayers operate at lower frequencies. In contrast, acoustic lenses offer a broader operational bandwidth with their sound intensity significantly enhanced; however, they currently have a relatively high threshold of lower frequency. In summary, when designing a high‐intensity low‐frequency acoustic wave generation system, the advantages of various structures should be considered comprehensively. Multiple structures can be combined to achieve better manipulation effects such as arranging plasmacoustic metalayers at the sound outlet of high‐intensity transducers and combining them with acoustic lenses to achieve multi‐stage amplification of sound intensity. However, this approach introduces complex structural designs and circuit connections. Therefore, how to achieve high‐intensity emission of low‐frequency acoustic waves remains an urgent problem to be solved.

## 4 Directional Control Of Low‐Frequency Acoustic Waves

### 4.1 Difficulties and Challenges of Low‐Frequency Acoustic Directional Control Technology

The directional emission of low‐frequency acoustic waves has long been a major challenge in the field of acoustic engineering, especially in the ultra‐low frequency range (typically between 20 Hz and 200 Hz). Due to the long wavelengths of low‐frequency acoustic waves, which are usually several to tens of meters, and even up to tens of meters for underwater low‐frequency acoustic waves, they exhibit strong diffraction properties, making directional control difficult. Generally, to achieve effective directional emission, the size of the acoustic device should be at least comparable to or larger than the wavelength. This requires the emitter to have a considerable physical size, which is often difficult to achieve in practical applications. With the development of materials science; phononic crystals^[^ ^14^, ^15^ ^]^ and acoustic metamaterials^[^ ^16^ ^]^ have been gradually developed for acoustic wave control. They can precisely control the propagation and focusing of acoustic energy at subwavelength and even deep subwavelength scales, especially for portable or space‐constrained scenarios.

**Figure 6:**
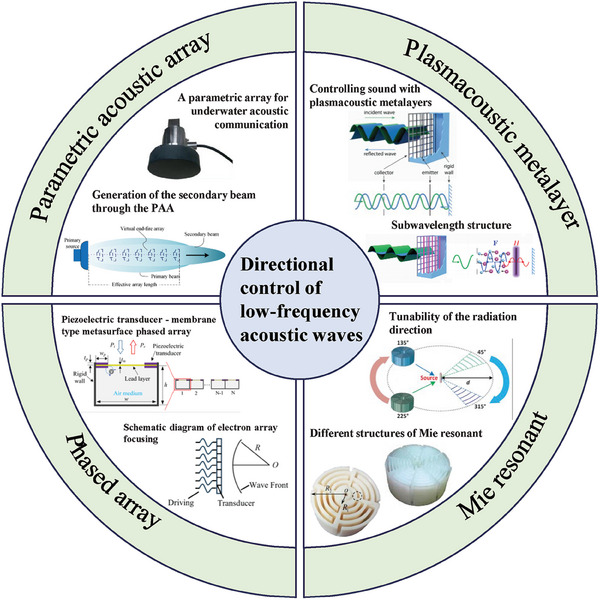
Various low‐frequency acoustic directional control technologies. 1) Parametric acoustic array. Reproduced with permission.^[^ ^99^, ^100^ ^]^ Copyright 2018, Elsevier and Copyright 2020, MDPI. 2) Phased array. Reproduced with permission.^[^ ^101^ ^]^ Copyright 2020, IOP Publishing. 3) Mie resonant. Reproduced with permission.^[^ ^38^, ^102^ ^]^ Copyright 2023, Springer Nature. 4) Plasmacoustic metalayer. Reproduced with permission.^[^ ^28^ ^]^ Copyright 2023, springer Nature.

Despite these challenges, various methods for directional emission of low‐frequency acoustic waves have been proposed. Some researchers focus on sound source arrays, employing acoustic metamaterials, parametric acoustic arrays, and multi‐source coordinated emission technologies to overcome the challenges of directional emission of low‐frequency acoustic waves; others utilize external devices with subwavelength structures to achieve directional control of acoustic waves, such as Mie resonators. Through precise structural design and intelligent signal processing, these technologies can enhance the directionality of low‐frequency acoustic waves to some extent. **Figure** **6** shows some of the common technologies for directional control of low‐frequency acoustic waves currently. This review will provide a detailed introduction to the research status of these technologies, allowing for a comparison of their advantages and shortcomings and offering some suggestions for future directional emission/radiation control of low‐frequency acoustic waves.

### 4.2 Methods for Directional Control of Low‐Frequency Acoustic Waves

A typical technique for directional emission of low‐frequency acoustic waves involves generating acoustic waves using acoustic speakers; and then, achieving a certain degree of directional emission through external devices such as horns or other sound emission apparatus. For example, aiming at the problem that low‐frequency acoustic signals have long wavelengths and are not easy to converge to form beams in a limited space, Tao et al.^[^ ^103^ ^]^ discussed and analyzed the methods of the multi‐sound source array, concave mirror energy convergence, and horn energy convergence, respectively, and the focused radiation performance of each method was verified by simulation tests. The simulation results indicated that the array configurations could realize the shaped pointing and beam control in the far field, but require the spacing between array elements to be half the wavelength of the corresponding signal, which would lead to an excessively long array and difficulty in frequency modulation. The concave mirror is large in volume and has a poor energy concentration effect, making it less practical; The acoustic horn can effectively gather infrasound signals across multiple frequency points, making it more suitable for practical application scenarios. However, these methods all correspond to large equipment size and significant space occupancy. In addition, directional acoustic waves can also be achieved at the sound generation stage through methods such as parametric acoustic arrays. In these methods, some can achieve device miniaturization through material improvements. Currently, acoustic wave control methods based on phononic crystals are mainly concentrated in the middle to high‐frequency bands. For example, to explore the simple low‐loss operation of directional acoustic transmission technology, Tang et al. studied the propagation of acoustic waves in resonant phononic crystals, and a transmission model based on acoustic metamaterials was constructed.^[^ ^104^ ^]^ Therefore, this section will focus on the application of novel acoustic metamaterials in the directional control of low‐frequency acoustic waves, spanning from several hundred hertz down to frequencies as low as 20 Hz.

#### 4.2.1 Parametric Acoustic Array

A viable approach to the directional emission of low‐frequency acoustic waves involves the use of parametric acoustic arrays (PAA). The discovery of the parametric array effect can be traced back to the 1960s.^[^ ^105^ ^]^ Westervelt^[^ ^106^ ^]^ first observed that low‐frequency waves (also known as difference‐frequency waves) were generated through the interaction of high‐frequency waves (also known as primary‐frequency waves). This technology utilizes nonlinear acoustic effects to produce a highly directional acoustic beam. Due to the nonlinear effect of the medium, the acoustic waves propagating in the same direction form difference‐frequency waves, as well as sum‐frequency waves and harmonic waves, and so on, because the attenuation of acoustic waves in the propagation process is proportional to the square of the frequency, the sum‐frequency, and harmonic waves attenuate more quickly, allowing it to produce highly directional difference‐frequency acoustic waves in the far field, as shown in Figure 6. The very high directivity of the acoustic beam, which enables long‐distance directional transmission, is a crucial feature of the parametric array.

Subsequently, Yoneyama et al.^[^ ^107^ ^]^ introduced the parametric array loudspeaker (PAL), which can generate directional audio beams in the air by amplitude‐modulating high‐intensity ultrasonic carrier signals with audible acoustic signals. PAL is an application of the acoustic parametric array in air, characterized by several ideal properties, such as narrow beamwidth across a wide frequency range and low acoustic attenuation over long distances.^[^ ^108^ ^]^ Compared to conventional speakers and broadside loudspeaker arrays, PAL also has the advantage of transmitting an equally narrow acoustic beam using smaller driving units. PAL employs two principal strategies for directional sound control: mechanical control methods and signal processing methods. Mechanical methods are constructed with motorized rotation platforms and reflective objects; while, signal processing methods are implemented in analog circuits and digital processors. The disadvantage of mechanical methods is their large overall size and complex structure, which makes control inconvenient. Although signal processing methods provide flexible control, they also introduce complex circuit connections.^[^ ^109^ ^]^

**Figure 7:**
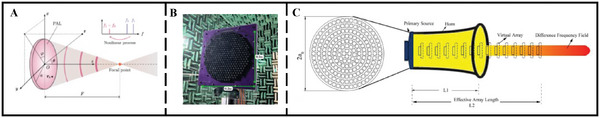
Focusing parametric array loudspeaker. A) Schematic sketch and B) the prototype of a 37‐channel circular focusing PAL with a radius of 0.1 m consisting of 367 circular ultrasonic emitters with a radius of 5 mm. Reproduced with permission.^[^ ^110^ ^]^ Copyright 2013, ASA. C) Schematic diagram of a horned parametric acoustic loudspeaker with a typical array of piezoelectric transducers. Reproduced with permission.^[^ ^112^ ^]^ Copyright 2018, ASME.

Although the acoustic parametric array has good directional effects at low frequencies, its energy efficiency is relatively low. To address this, Zhong et al.^[^ ^110^ ^]^ utilized a parametric array loudspeaker to focus the acoustic field, constructing a prototype of a 37‐channel focusing PAL, achieving directional focusing of acoustic waves as low as 250 Hz and improving the poor low‐frequency response without focusing of conventional PAL, as shown in **Figure** **7**A,B. However, while this approach effectively focuses acoustic waves in designated directions, the amplification of sound intensity it permits is inherently constrained, and it is accompanied by significant energy dissipation. To improve the conversion efficiency of PAA devices, Sayin and Guasch^[^ ^111^ ^]^ assembled an ultra‐megaphone with a parametric loudspeaker and a horn. On the one hand, the ultra‐megaphone can overcome the cutoff issues present in conventional exponential horn loudspeakers. On the other hand, the horn can improve radiation efficiency and enhance the conversion efficiency of PAA devices. In addition, Tong et al. proposed an analytical model for the difference frequency acoustic pressure generated by a horned PAL and verified that a horn can make a significant impact on the difference frequency acoustic pressure and the directivity effect generated by a PAL, as shown in Figure 7C.^[^ ^112^ ^]^ The performance of a horned PAL can be improved by an appropriate selection of control parameters to produce highly directional acoustic waves compared to conventional types of PAL.

As mentioned above, the most remarkable acoustic characteristic of the parametric acoustic array is its sharp directivity at low frequencies, which also plays a significant role in noise control^[^ ^113^, ^114^ ^]^ and acoustic field analysis.^[^ ^115^ ^]^ In addition, the acoustic characteristics of the parametric array in water are essentially the same as those in air, with sharp directivity at low frequencies and the ability to form broadband beams with virtually no sidelobes.^[^ ^116^ ^]^ Therefore, PAA is highly suitable for high‐resolution detection of subsea strata profiles and represents an important development direction for seabed profiling measurements.^[^ ^100^ ^]^

Further, Kang et al. extended the study of directional emission of acoustic waves using acoustic parametric arrays to the infrasonic frequency range.^[^ ^117^ ^]^ By combining MATLAB and finite element analysis software based on nonlinear acoustics, they verified the high directivity of infrasonic waves. They first modulated the infrasonic waves to be emitted, carrying them on an ultrasonic carrier; and then, used a transducer array to send the modulated ultrasonic waves into the air medium. As a result, during their propagation, the original infrasonic signal was continuously demodulated through nonlinear interactions. As the attenuation of acoustic waves is proportional to the square of the frequency, the high‐frequency ultrasonic wave signals would rapidly attenuate during propagation, leaving only the continuously demodulated infrasonic waves to be accumulated and superimposed forward, thereby achieving a highly directional infrasonic source array.

Nevertheless, the limited energy conversion efficiency of parametric arrays, coupled with the challenges in receiver alignment, has resulted in a paucity of reported engineering applications. In addition, for the directional control methods of infrasonic waves using the aforementioned parametric acoustic arrays, on the one hand, parametric acoustic arrays require two high‐frequency acoustic sources (usually ultrasonic waves) to generate infrasonic waves. To obtain infrasonic waves with lower frequencies, the frequency difference between the high‐frequency sources needs to be small, which means very precise and stable high‐frequency sources are required. Further, the selection of transducers has a significant impact on the performance of PAA. For the one thing, the transducer array should have a large surface area to ensure high emission power and high sound source level. For another, Rayleigh's limit law indicates that low‐frequency sonar requires large‐aperture transducers to achieve fine directivity, which increases the size of the sound source; on the other hand, as the generation of infrasonic waves relies on the nonlinear interaction of high‐frequency acoustic waves, this may lead to low energy conversion efficiency and require higher emission power to produce sufficiently strong infrasonic waves. Consequently, it is necessary to find more effective methods to improve the transmission efficiency of high‐intensity acoustic wave emission in the infrasonic frequency range.

#### 4.2.2 Phased Array

A phased array is a technology that electronically controls the direction of a beam. It achieves precise beam steering by adjusting the phase or amplitude of individual elements within the array. Phased array technology is widely used in radar, communication, navigation, and other fields, characterized by high reliability, versatility, and adaptability. Phased array systems can employ digital beamforming techniques to rapidly scan and locate beams. However, while phased arrays exhibit directionality, they also produce sidelobes, which can vary significantly in form depending on the array elements and configurations. The directional effect of phased arrays is primarily measured by two parameters: the directional effect itself and the width of the main lobe.

**Figure 8:**
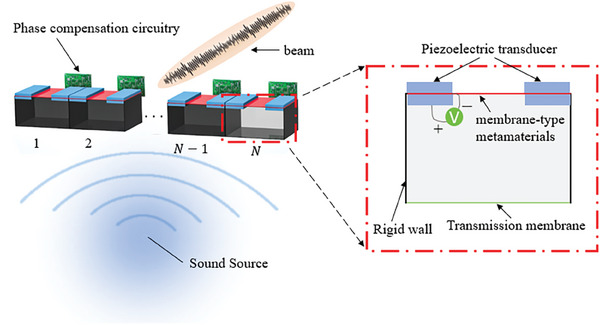
Directional emission scheme for acoustic waves transmitted from the bottom of an array composed of membrane‐type metasurface and piezoelectric transducer units.

Currently, acoustic control technology based on phased arrays is mainly focused on the high‐frequency range,^[^ ^118^ ^]^ and the sound sources are primarily ultrasonic waves, which inherently have strong directivity. Therefore, the application of phased array technology can well meet the requirements. For instance, in the medical field, the use of phased array‐focused ultrasound enables non‐invasive tissue ablation for patients,^[^ ^119^ ^]^ and in the field of ultrasound imaging,^[^ ^120^ ^]^ among other rich applications. In contrast, low‐frequency acoustic waves have longer wavelengths and stronger penetration, especially infrasound, which is a spherical wave and more difficult to achieve directional emission. Therefore, there is little research on low‐frequency acoustic directional emission technology based on phased arrays. Novel phased array techniques are being investigated to enable directional manipulation of low‐frequency acoustic waves. For instance, Song et al.^[^ ^121^ ^]^ designed a novel parabolic phased array system with non‐uniform spacing for infrasonic focusing. By combining theoretical analysis with computer simulation and utilizing an optimized pseudo‐inverse matrix algorithm, they thoroughly investigated the effects of various spatial structural parameters of the infrasonic array on focusing performance. MATLAB simulation results indicate that, compared to planar phased arrays, this system offers superior directional focusing capabilities for infrasound and higher acoustic intensity gain, making it an ideal sound source array. In addition, Li et al. studied the structural designing of array elements, combining membrane‐type acoustic metasurface with piezoelectric transducers.^[^ ^122^ ^]^ They used piezoelectric transducers to adaptively control the tension, vibration frequency, and other physical parameters of the acoustic metasurface to adjust its acoustic impedance, thereby influencing the reflection or transmission coefficients of incident acoustic waves. By combining multiple array elements, they formed a phase gradient in the outgoing acoustic waves, thereby achieving directional control of low‐frequency acoustic waves, as shown in Figure 6. This method allows the manipulation of acoustic waves within subwavelength structures. However, as the frequency decreases and the wavelength of the acoustic wave becomes longer, its penetration ability increases, making it extremely difficult to achieve acoustic wave reflection using this technology. This is due to the piezoelectric transducers making it challenging for the vibration frequency of the membrane‐type acoustic metamaterial to match that of the incident acoustic wave. It is feasible to emit extremely low‐frequency acoustic waves from the base of the structure and employ the metasurface of the membrane to adjust its refractive index, in coordination with the phase gradient produced by the array, to achieve directional control of the acoustic wave, as illustrated in **Figure** **8**. Similar to the research on piezoelectric metasurfaces, Chen et al.^[^ ^123^ ^]^ explored a convenient tunable metasurface Helmholtz resonator design, where moving sliders allow for full phase shifts with high transmission ratios across a wide bandwidth. Further, based on arrays composed of asymmetric Mie resonance elements, Lan et al.^[^ ^124^ ^]^ employed a mechanical rotation method to adjust the phase of acoustic waves, thereby achieving the purpose of controlling the direction of acoustic waves.

For traditional phased array technology, due to the extremely long wavelength of very low‐frequency acoustic waves (particularly underwater), the phased array requires a very large physical size and highly precise phase control. The challenge lies in how to use deep subwavelength acoustic metamaterial arrays to achieve mechanical rotation or electronic adjustment of excitation signals to complete phase shifts within a 2π range. In addition, an increase in the number of phased array elements contributes to a narrower spatial beam and more concentrated energy. However, this also significantly increases the system's complexity and energy consumption. Therefore, it is necessary to comprehensively consider and explore the effects of array layout and the number of array elements on performance parameters such as spatial resolution, beam width, energy consumption, and reduction of main beam sidelobes.

#### 4.2.3 Mie Resonant

Owing to the breakthroughs in metamaterials, substantial progress has been made in recent years in overcoming diffraction limitations for directional transmission at deep subwavelength scales.^[^ ^125^ ^]^ Conventional methods for controlling the direction of acoustic waves mainly rely on adjusting the sound source (such as using phased array arrangements and Bessel beam patterns) and utilizing the anisotropy, anti‐resonance, diffraction, and band‐edge states of phononic crystals.^[^ ^15^ ^]^ However, these methods have disadvantages such as large volume, expensive materials, complex structures, and lack of versatility. As the wave manipulation characteristics of Mie resonance depend solely on the microstructural parameters of the material itself, it offers greater flexibility, tunability, and potentially higher gain compared to phononic crystals and multi‐microstructured units that require spatial arrangements.

In the field of directional control of acoustic waves, the factors influencing the directionality of acoustic waves have been identified as the radiation area of the acoustic source, the structure of the radiation surface, and the frequency of the acoustic wave. The directional control of low‐frequency acoustic waves, especially infrasound, has long been a blind spot in research. Directional control of low‐frequency acoustic waves can be achieved by altering the boundary conditions of the medium and structural configurations.^[^ ^126^ ^]^ The directional propagation of acoustic waves using Mie resonant is classified as a form of structural control. As shown in Figure 6, by adjusting the geometric parameters of the Mie resonance unit microstructure, such as the number of bends and channel width, precise control over acoustic wave transmission can be achieved while maintaining the size of the structure to be much smaller than the wavelength of the acoustic wave, that is, at deep subwavelength scales.^[^ ^36^ ^]^ Therefore, Mie resonant is of great significance for achieving the miniaturization of acoustic devices.

**Figure 9:**
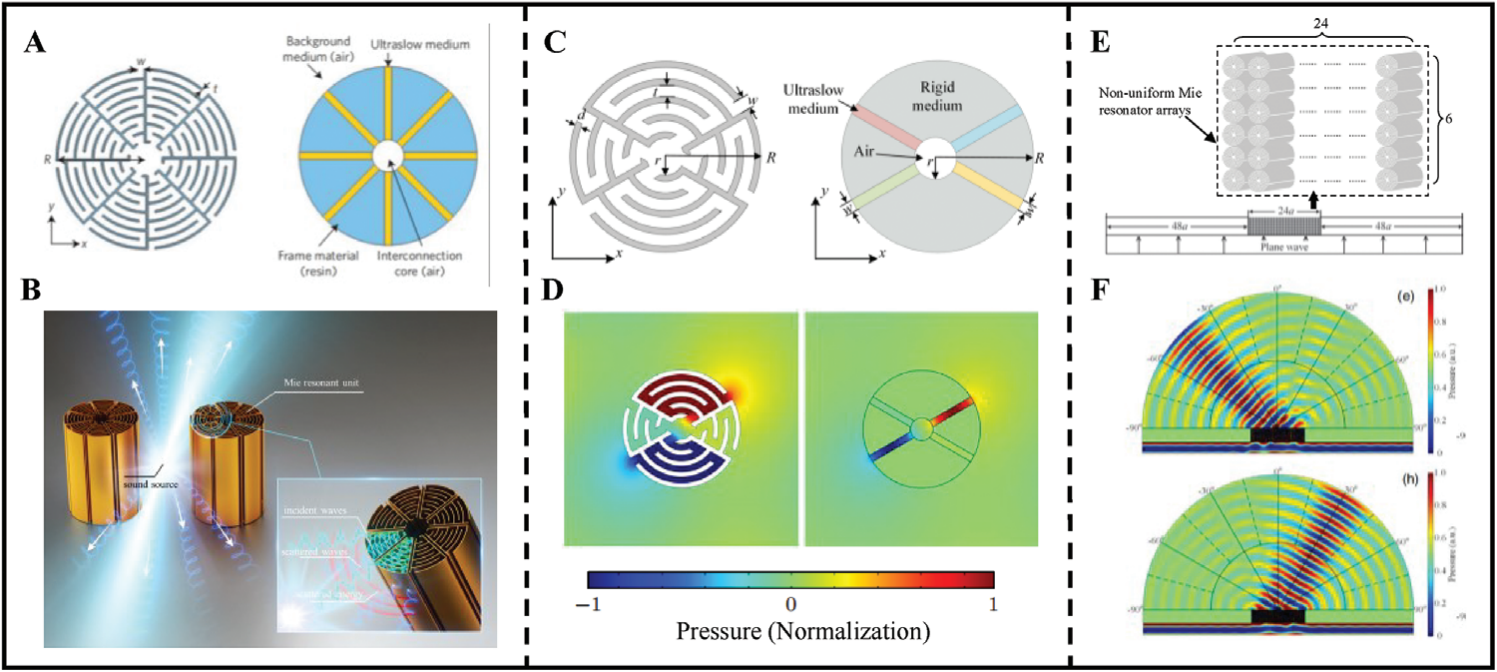
Acoustic wave directional control based on Mie resonance. A) Cross‐sectional view of a cylindrical labyrinth structure (left) and microstructure equivalence model (right). Reproduced with permission.^[^ ^38^ ^]^ Copyright 2015, Springer Nature. B) Renderings of the acoustic wave directional control of the Mie resonator. C) Cross‐sectional view of a cylindrical non‐uniform labyrinth structure (left) and microstructure equivalence model (right). D) Dipole mode and dipole mode sound pressure field plots of a cylindrical non‐uniform labyrinth structure. Reproduced with permission.^[^ ^127^ ^]^ Copyright 2022, AIP Publishing. E) Rotatable non‐uniform Mie resonator arrays and F) directionally controlled simulated acoustic pressure field. Reproduced with permission.^[^ ^124^ ^]^ Copyright 2022, IOP Publishing.

Mie resonance units can be realized through the construction of multi‐layer structures. Cheng et al. proposed the use of low‐effective acoustic velocity effects in highly symmetric folded space structures to create fluid micro‐units with ultra‐slow acoustic velocities.^[^ ^38^ ^]^ **Figure** **9**A shows cross‐sectional views of the folded space structure in the *x*–*y* plane (left) and the equivalent model of the microstructure (right). The labyrinthine structure with a radius *R* (much smaller than the wavelength) is divided into eight channels, each consisting of folded space with a width *w* and a number of folds *N*. In each channel, the hard boundary frame forms a pathway that causes the acoustic wave propagation to reach the center of the structure with a path length several times that of the original channel. As the size of the Mie resonant is much smaller than the wavelength, in the long‐wavelength approximation, the distance is constant, the time is prolonged, and the effective speed of the acoustic wave from the outside to the inside is slowed down. This results in a structure with a high relative refractive index *n*_r_. The magnitude of the refractive index is determined by the ratio of the total acoustic path length to the unit size, which is proportional to the number of folds *N*; thus, allowing the refractive index to be controlled by changing the size of the elements and the number of folds. The right side of Figure 9A shows the equivalent physical model of the structure, which consists of interconnected air regions (white) separated by resin frame material (blue). Monopole Mie resonance is excited when the acoustic waves in the eight channels are in phase; while, dipole Mie resonance and other multiple Mie resonances can be excited when the eight channels are out of phase. By adjusting the parameters of the Mie structure to control whether resonance occurs, it is possible to effectively enhance or suppress acoustic waves at specific frequencies. Therefore, while achieving directional control of acoustic waves, the Mie resonance structure can also achieve a certain degree of acoustic focusing to increase acoustic energy. Previous studies have verified the ability of Mie resonance structures to amplify and receive signals at subwavelength scales. Jia et al.^[^ ^39^ ^]^ proposed a subwavelength bianisotropic hybrid Mie resonator (BHMR); the acoustic resonances and Willis couplings of BHMR together contribute to the enhanced monopole and dipole sound antennas. It mainly uses the Willis coupling effect to make the sound waves excited by the central monopole sound source in the structure. The interaction and coupling of the pressure field and the velocity field are generated internally, resulting in the opposite phase of the sound field in the upper and lower parts of the BHMR; and then, a radiation pattern with obvious directivity is formed in space, mainly radiating in the horizontal direction, thereby realizing the directional radiation of acoustic waves. In 2017, Lu et al. utilized the interaction between Mie resonance units and the non‐directional point sound source to achieve bidirectional radiation of acoustic waves and effectively control acoustic waves at subwavelength scales.^[^ ^36^ ^]^ As shown in Figure 9B, the Mie resonance units on both sides of the point sound source effectively block the propagation of acoustic waves in the direction of the two structures' connection; while, enhancing the radiation energy in the vertical direction, thereby effectively achieving bidirectional radiation of acoustic waves. Based on the symmetrically folded space structure, Zhang et al. applied Mie resonance structures to the design of tunable acoustic antennas, allowing the directionality of the antenna to be adjusted freely to rotate and direct acoustic radiation within the entire 2π range, as shown in Figure 6. They utilized tunable acoustic antennas to achieve a 2.33‐fold enhancement in radiation power and an 8.93‐fold enhancement in sound intensity, indicating that the antenna has a significant ability to concentrate acoustic energy in the main beam direction. It has been demonstrated that Mie resonance can enhance radiation power and sound intensity and achieve specific directional acoustic wave enhancement and sidelobe suppression.^[^ ^87^ ^]^ In addition, Zeng et al. designed a Mie–Helmholtz structure (M–H structure) based on Mie resonance, which can further enhance far‐field SPL and unidirectional directivity by utilizing the high sound pressure inside the central cavity.^[^ ^37^ ^]^

In the study by Zhang et al.,^[^ ^87^ ^]^ mechanical rotation of the Mie resonance units was used to achieve directional scanning of acoustic waves at subwavelength scales. However, this method requires moving the entire Mie resonance unit to scan acoustic waves within a 2π range, which is very inconvenient in practical applications. Moreover, the acoustic properties of commonly used Mie resonance elements with uniform geometric shapes do not change when rotated around the center. In other words, acoustic waves can only propagate in a fixed direction, and dynamically adjustable acoustic directional radiation cannot be achieved. Liu et al.^[^ ^128^ ^]^ designed a cylindrical heterogeneous labyrinthine structure,^[^ ^129^, ^130^, ^131^ ^]^ which can be used to dynamically switch directional radiation of acoustic waves in different directions by utilizing the characteristics of dipole dynamic switching and rotational anisotropy of the microscopic structures, as shown in Figure 9C. By combining the directional response characteristics of dipole resonance with the rotational anisotropy of the structure, when the structure is in the dipole resonance mode, the nonuniform labyrinthine structure rotates around its center, and the position and direction of the acoustic waves radiated outward by the microstructures change. Figure 9D shows the acoustic field diagram of the nonuniform resonator in the dipole mode, from which it can be seen that the acoustic waves are directionally radiated through two large‐area fan‐shaped regions. In addition, by compactly arranging the nonuniform labyrinthine structures on a ring outside the point sound source and dynamically changing the rotation angles of multiple microstructures, the purpose of dynamically controlling the direction of acoustic directional radiation can be achieved.

Further, Lan et al.^[^ ^124^ ^]^ proposed a phased array system based on rotatable non‐uniform Mie resonance units, which can scan in any direction by mechanically rotating the Mie resonance array according to the generalized Snell's law and adjusting the phase shift distribution along the metamaterial, as shown in Figure 9E. This method provides a mechanically controlled phased array that achieves directional control of acoustic waves, enabling high‐intensity low‐frequency acoustic wave manipulation at subwavelength scales. However, the rotation of the Mie resonance array inevitably introduces additional mechanical control equipment, which is complex and slow to respond and is not as flexible, convenient, and fast as conventional electronically adjustable phased arrays. Therefore, it is necessary to reasonably design the rotation control structure and develop corresponding control systems, making it suitable for practical applications.

Mie resonance induces acoustic wave resonance within the metamaterial, thereby achieving directional radiation and energy focusing of acoustic beams. This method can also achieve directional control of low‐frequency acoustic waves at deep subwavelength scales. As the resonance frequency is closely related to the size of each structure, directional radiation of infrasound can be achieved through reasonable parameter design. By combining simulation and experimentation, the optimal Mie resonance units and array unit arrangements for the infrasonic frequency band can be obtained.

#### 4.2.4 Plasmacoustic Metalayer

Plasmacoustic metalayer is an advanced technology that uses plasma techniques to control and manipulate the propagation of acoustic waves. This technology combines concepts from plasma physics and acoustic metalayers, achieving directional emission and manipulation of acoustic waves by introducing specific structures or patterns into the plasma.

Sergeev et al. demonstrated that the dynamics of thin‐layer air plasma can be controlled in an ultra‐broadband manner and can interact with sound over deep subwavelength distances, as shown in Figure 6. Utilizing the unique physical properties of plasmonic acoustic layers, the experiment demonstrated perfect sound absorption and tunable sound reflection, ranging from several Hz to kHz, with the thickness of the transparent plasma layer being as low as *λ*/1000, where *λ* is the wavelength of the acoustic waves. Various applications require this bandwidth and compactness, including noise control, audio engineering, room acoustics, imaging, and metamaterial design.

The process of directional control of low‐frequency acoustic waves using plasma acoustic metamaterials is relatively straightforward. By adjusting the amplitude and phase of the external high‐voltage excitation source, as well as the distance between the collector (grounded metal mesh) and the emitter in the corona discharge device, the acoustic impedance of the medium surrounding the plasma acoustic metamaterial can be altered. This modification regulates the amplitude and phase of the reflected wave. The reflected wave coherently superimposes with the original wave in the desired direction; while, canceling and attenuating in other directions, thereby achieving directional control of the acoustic wave. However, the generation of plasma is highly susceptible to environmental parameters such as air humidity, ambient pressure, and temperature. Significant changes in these parameters due to variations in gas mixtures and molecular energy alter the ionization process. This affects the shape of the discharge voltage–current characteristics, thereby impacting the dynamic performance of the plasma acoustic metamaterial for acoustic control and leading to unstable behavior in the control system. Therefore, it is recommended to incorporate external devices, such as temperature and humidity sensors, and pressure sensors, to monitor environmental changes when using corona discharge equipment. Based on these parameters, feedback control should be applied to the system to adjust the excitation source promptly, ensuring the efficient and stable generation of plasma.

**Figure 10:**
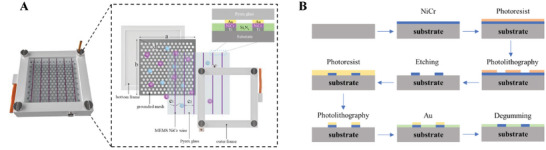
A) Design of acoustic reflector based on MEMS corona discharge. B) MEMS manufacturing process based on NiCr corona discharger.

Moreover, the directional control technology of acoustic waves based on plasmacoustic metalayers holds great potential for applications, enabling the reflection of acoustic waves by extremely thin unit layers and their interference with the original acoustic waves, thereby completing the directional control of the final radiated acoustic waves. This technology is primarily used to enhance the sound intensity of low‐frequency acoustic waves. To control the amplitude and phase of acoustic waves in the infrasonic frequency range, it is necessary to increase the concentration and uniformity of the plasma. When using corona discharge devices to generate plasmonic acoustic layers to control the acoustic parameter characteristics of the surrounding medium, micro–electro–mechanical system (MEMS) technology can be employed to manufacture NiCr thin film discharge devices, achieving efficient and uniform plasma generation, as shown in **Figure** **10**. In addition, the structure, shape, and other external factors of the corona discharge device should be considered for their impact on acoustic control. A comprehensive design should be developed for a plasmacoustic metalayer prototype that can achieve adjustable amplitude and phase of infrasonic waves, high sensitivity, convenient control, and wide bandwidth.

**Figure 11:**
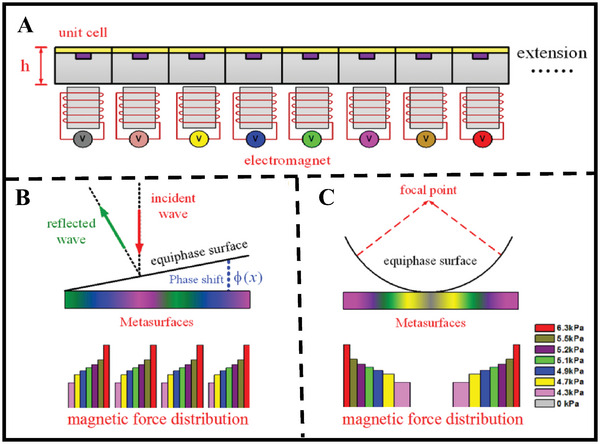
A) Schematic diagram of the proposed voltage‐controlled magnetic metasurface, with different colors representing different input voltages. Schematic diagrams of B) anomalous reflection and C) focusing. The bar graphs show the desired magnetic force distributions for achieving related functionalities at 400 Hz. Reproduced with permission.^[^ ^136^ ^]^ Copyright 2017, Springer Nature.

In addition, researchers have been exploring various methods to achieve directional control of low‐frequency acoustic waves. On the one hand, they have expanded on existing technologies and materials to provide new ideas for achieving directional propagation of low‐frequency acoustic waves. For example, Yuan et al. utilized bent waveguides to achieve efficient directional transmission of acoustic waves over a wide frequency range.^[^ ^132^ ^]^ Udawalpola et al. studied acoustic horns that can achieve very uniform far‐field directivity patterns but with significant efficiency loss. Therefore, to obtain more precise control over far‐field directivity without sacrificing too much efficiency, they proposed combining acoustic horns with acoustic lenses to achieve better acoustic directionality and energy focusing.^[^ ^133^ ^]^ Ding et al. proposed using a finite array of dipole sources to enhance the directionality and radiation gain of sound. By implementing dipole modes with Helmholtz resonators, the dipole array exhibits better directionality and smaller sidelobes over a wider frequency range compared to finite point acoustic source arrays.^[^ ^134^ ^]^ Further, in addition to the aforementioned acoustic metamaterials, Qi et al. proposed a polygonal acoustic metasurface for acoustic reflection focusing and energy confinement. The more edges the polygon has, the stronger the focusing capability. Due to its variable structural design and powerful acoustic focusing ability, this structure exhibits excellent performance in acoustic energy confinement under various working conditions.^[^ ^135^ ^]^ Chen et al. introduced a membrane‐type acoustic metasurface with a deep subwavelength thickness (≈1/85*λ*), which is crucial for guiding low‐frequency acoustic waves. To overcome the limitation of a narrow working band, a magnetically tunable mechanism was developed for the metasurface, as shown in **Figure** **11**. By utilizing magnetic fields to customize phase distributions, acoustic wave redirection and focusing can be demonstrated over a wide bandwidth.^[^ ^136^ ^]^

Further, acoustic metasurfaces can be used to manipulate underwater acoustic waves. Compared to applications in the air, underwater structures are more likely to achieve impedance matching, and the assumption of hard boundaries is often invalid.^[^ ^137^ ^]^ Chen et al. designed a novel resonator‐based metasurface to control the reflection of underwater acoustic waves. Each metasurface unit consists of an aluminum plate and a lead plate. By adjusting the thickness of the plates, a full 2π phase shift of the reflected wave can be achieved. Acoustic waves can be directed and focused at frequencies as low as 500 Hz.^[^ ^138^ ^]^

On the other hand, researchers have also explored how to achieve faster and more accurate directional emission of acoustic waves by starting with algorithms in software. For instance, in phased arrays, rapid beamforming is crucial for the directional transmission of acoustic waves. The essence of beamforming is to enhance the emission directivity of the beam in space. By weighting and filtering the signals received by each unit, output in a certain direction is obtained, that is, the array gain is concentrated in a desired direction, achieving beam focusing. However, in practical applications, the situation is very complex with many interference factors, especially in underwater environments, which include irregular terrain, various reflections and scattering phenomena, and multipath effects. In such environments, the performance of traditional beamforming algorithms may be limited. Accordingly, emerging artificial intelligence algorithms such as machine learning and deep learning models can be leveraged to learn and adapt to the complex changes in underwater environments, dynamically adjust beamforming parameters, and optimize the directional transmission performance of low‐frequency acoustic signals.^[^ ^139^ ^]^ Artificial intelligence algorithms can achieve multi‐target tracking and recognition through pattern recognition and classification techniques, accurately distinguish different targets, and provide corresponding beamforming strategies, offering strong technical support for precise directional transmission. Artificial intelligence algorithms, especially deep learning technology, can efficiently process large‐scale data, perform real‐time analysis and processing, improve the system's response speed and accuracy, and achieve autonomous decision‐making and optimization, enhancing the system's performance in different tasks and complex environments.

### 4.3 Comparison of Various Low‐Frequency Acoustic Directional Control Methods

The above contents provide a relatively detailed review of various methods for directional control of low‐frequency acoustic waves. This includes conventional approaches to improving acoustic wave generation, such as parametric acoustic arrays and phased arrays. In addition, it covers methods using novel phononic crystals and acoustic metamaterials for directional control of acoustic waves, such as Mie resonators and plasmacoustic metalayers. The principles of these technologies, along with their advantages and disadvantages, are compared as shown in **Table** **2**.

**Table 2:** Comparison of different directional control techniques.

Technical solution	Advantage	Disadvantage	Frequency/bandwidth	Beam directivity	Refs.
Parametric acoustic array	• Low frequencies with high directivity • Small size • Very small sidelobes	• Low radiated power • Large energy loss • Complexity in design	5 kHz^[^ ^112^ ^]^	$8.7^{\circ }$ (58 dB @ 3 m)^[^ ^112^ ^]^	[43, 100, 116, 140]
Phased array	• Strong adaptability • Convenient control • Sub‐wavelength scale	• A large number of array elements • Complex signal‐processing techniques	781.5 Hz^[^ ^141^ ^]^	$22.33^{\circ }$ [141]	[122, 123]
Mie resonant	• Low energy loss • Sub‐wavelength scale	• High requirements for material stiffness and toughness • Narrow bandwidth	1360 Hz^[^ ^36^ ^]^	$\pm 22^{\circ }$ (−3 dB)^[^ ^36^ ^]^	[36, 87, 124]
Plasmacoustic metalayer	• Small characteristic size • High reflection efficiency	• An external high‐voltage source is required	20–2000 Hz^[^ ^28^ ^]^	–	[27, 28]

Among these, parametric acoustic arrays and phased arrays, which employ active methods to control and modify sound sources, have the advantages of flexible manipulation and superior directional performance. However, they also face issues such as the need for a large number of electronic components and complex circuits. Therefore, there is a need to develop a new, efficient passive directional sound emission technology. It has been demonstrated that by using phononic crystals to alter the radiation of sound sources, high directionality and radiation enhancement can be achieved.^[^ ^14^ ^]^ However, this method applies to relatively high‐frequency acoustic waves, and there have been no reports so far of using this material to achieve directional control of acoustic waves in the frequency range of hundreds of hertz. In addition, acoustic metamaterials are artificial materials used to manipulate acoustic waves, possessing characteristics that are difficult to achieve with conventional natural materials, such as negative mass density, negative bulk modulus, double‐negative metamaterials, and extreme anisotropy. Based on these properties, metamaterials can manipulate acoustic waves within a wavelength range to produce various functions. As a type of acoustic metamaterial, acoustic metasurfaces,^[^ ^72^, ^142^ ^]^ due to their ultra‐thin planar structure and fully controllable phase delay, provide a powerful capability for achieving directional sound emission. Based on the generalized Snell's law,^[^ ^122^ ^]^ the direction of emitted acoustic waves can be flexibly manipulated by adjusting their phase distribution. However, for low‐frequency acoustic waves with long wavelengths, the sound source can be considered as a point source, which poses a significant challenge to directional radiation. To overcome this issue, subwavelength Mie resonances with high relative refractive indices are introduced, enabling efficient directional acoustic radiation from point sources, and the radiation direction can be controlled by rotating the Mie resonances.^[^ ^87^ ^]^ Nevertheless, these units are specific Mie resonance structures with fixed operating bandwidths; so, directional acoustic radiation can only operate within a very narrow band or even within a single frequency range, which severely hinders their practical applications. Acoustic metamaterials based on resonance and gradient impedance anisotropy can also achieve directional sound emission enhancement. However, how to further increase the effective working bandwidth remains an unresolved issue. Although plasmacoustic metalayers can achieve acoustic focusing and a certain degree of directional control at deep subwavelength scales, their ability to reflect acoustic waves is limited, especially in the low‐frequency bandwidth, where a huge external voltage input is usually required to barely cope with long‐wavelength acoustic waves. Further, Table 2 also presents the optimal values, considering both directional radiation angles and operational frequencies for various acoustic wave directional technologies. Among these, parametric acoustic arrays offer the best directional angles but operate at higher frequencies. In contrast, phased arrays and Mie resonances have lower operational frequencies and directional radiation angles, but their working bandwidths still need improvement; plasmacoustic metalayers have a larger bandwidth, while their directional performance has not been validated. Therefore, achieving directional control of acoustic waves at very low frequencies and across multiple scenarios remains an urgent issue to be addressed.

## 5 Challenges and Perspective

The directional radiation of acoustic waves has long been a focal point of research in the field of acoustics. High‐frequency acoustic waves, due to their excellent directivity and resolution, are widely used in areas such as medical ultrasound and communication. However, the weak penetration and short transmission distance of ultrasound limits its application in marine environmental detection and long‐range communication. In contrast, low‐frequency acoustic waves can effectively compensate for these limitations. Due to their inherent acoustic properties, research on the directional emission of low‐frequency acoustic waves, especially those with high SPL, is relatively scarce. This article systematically reviews various methods for achieving high‐intensity and directional radiation of low‐frequency acoustic waves.

On the one hand, in existing technologies, as the frequency decreases, the maximum sound intensity that low‐frequency sound sources can achieve also gradually diminishes, posing challenges for the high‐intensity emission of low‐frequency acoustic waves. Low‐frequency acoustic waves exhibit slower energy attenuation compared to high‐frequency acoustic waves, allowing them to travel over great distances. However, within the low‐frequency range, energy tends to disperse in all directions, with most of the acoustic radiation energy unable to propagate to the far field, resulting in extremely low sound emission efficiency.^[^ ^37^ ^]^ Researchers are continuously exploring methods to enhance the energy of low‐frequency sound emission. For one thing, the performance of sound sources can be enhanced by utilizing acoustic resonators^[^ ^24^, ^26^ ^]^ and various specially designed sound generators or by employing energy harvesting techniques to improve their efficiency.^[^ ^143^ ^]^ However, these devices have significant bandwidth limitations, only enabling sound intensity enhancement at a specific frequency or within a very narrow frequency band. For another, external devices such as acoustic lenses and plasmacoustic metalayers are used to modulate the sound source, thereby achieving energy focusing and enhancement of the acoustic wave. These methods are characterized by flexible manipulation and the ability to achieve acoustic wave energy focusing at deep sub‐wavelength sizes. Nevertheless, issues such as bandwidth limitations and the introduction of complex control circuits persist.^[^ ^109^ ^]^

On the other hand, the increasing wavelength as the frequency of acoustic waves decreases makes it increasingly difficult to achieve acoustic wave reflection and phase manipulation. The directivity of current low‐frequency sound sources is always poor in open space; most acoustic sources with dimensions much smaller than the operating wavelength essentially exhibit non‐directional radiation patterns, emitting acoustic energy at wide angles in nearly all directions. Traditional methods to enhance directivity primarily rely on modifying the sound source, such as using large horns to restrict the radiation space of the sound source or employing phased arrays^[^ ^33^ ^]^ and Bessel beam patterns to generate wave interference.^[^ ^87^ ^]^ In recent years, with the emergence of acoustic metamaterials, the direction control of acoustic waves and the confinement of acoustic beams have been extensively explored. For instance, phononic crystals, acoustic metasurfaces, and dual‐cavity resonators can modify boundary conditions, resonances, or the entire propagation region to adjust the directionality of acoustic waves.

Another issue is that when it comes to the direction control of very low‐frequency acoustic waves and infrasound, the size of the entire system will increase significantly, limiting the miniaturization of the device. In addition, the strong penetration and high diffraction characteristics of low‐frequency acoustic waves also result in poor performance of these direction control devices. Though there has been research using high‐strength and high‐toughness nanomaterials to fabricate lightweight and flexible acoustic devices which have shown superior performance,^[^ ^144^ ^]^ there is still a significant gap in achieving the miniaturization of directional systems. Therefore, utilizing subwavelength‐scale devices to control the directional radiation of acoustic waves remains a challenging but promising research endeavor.^[^ ^145^ ^]^

Moreover, current acoustic wave directional emission technologies rarely involve frequencies in the tens of hertz range and below, and the application scenarios are single, with almost all research focused on sound systems in the air; while, applications in underwater environments are seldom explored. The main reason for this is that the speed of sound propagation underwater is about four times faster than in the air, resulting in extremely long wavelengths for low‐frequency acoustic waves underwater, making it more difficult for existing technologies to achieve directional propagation. In addition, the temperature, salinity, and depth of water, as well as waves and other dynamic conditions,^[^ ^146^ ^]^ also affect acoustic wave propagation, further increasing the difficulty of achieving directional radiation of low‐frequency acoustic waves. The existing acoustic wave steering technology is not very effective underwater, and traditional beamforming techniques show very limited performance in shallow water.^[^ ^147^ ^]^ Although Blomberg et al.^[^ ^148^ ^]^ proposed the use of adaptive beamforming techniques to dynamically adjust the beam direction based on real‐time feedback, this is only suitable for shallow water environments, and how to effectively control acoustic waves in more complex deep water environments remains to be studied. In addition, the existing underwater directional transducers can achieve relatively high frequencies, often above several kilohertz.^[^ ^149^ ^]^ Moreover, most of these transducers are made from conventional materials that only function effectively when the wavelength is much smaller than their size. In contrast, Norris^[^ ^150^ ^]^ has designed a phononic crystal plate composed of monometallic phases, which shows good focusing ability in water. Chen et al.^[^ ^151^ ^]^ demonstrated an ultra‐thin reflecting metasurface for manipulating underwater low‐frequency acoustic waves, which could achieve sharp focusing of incident waves over a wide frequency range of 20–800 Hz. However, the frequency of acoustic waves applicable to this research was still relatively high, and there remained a significant gap in achieving the manipulation of infrasound. In addition, Dong et al.^[^ ^86^ ^]^ proposed a deformation‐preserving transformation acoustic method inspired by toothed whales. Through the design and manufacture of acoustic devices containing air cavities, steel and gradient refractive index metamaterials, precise control of the direction and shape of underwater sound wave propagation was realized. The research used conformal mapping technology and the special structure of acoustic metamaterials to successfully convert sound waves emitted by omnidirectional sound sources into directional sound beams. Current research has demonstrated the multifunctional and potent manipulation capabilities of metamaterials for low‐frequency acoustic waves. Therefore, future research still needs to be based on metamaterials to achieve better directional radiation of low‐frequency acoustic waves.

In the development of future low‐frequency acoustic wave directional control technology, it is essential to fully integrate multiple disciplines, seeking new solutions from the development of high‐intensity sound sources to the control of acoustic wave propagation processes and the exploration of acoustic wave application potential in various fields. Specifically, in the development of acoustic systems, combining emerging materials with conventional technologies, such as utilizing acoustic metamaterials to miniaturize acoustic resonators and acoustic wave propagation control devices, is an excellent strategy. To achieve this technology, on the one hand, new materials need to be developed, exploring materials capable of efficient energy conversion and capable of withstanding high‐intensity resonance to meet the demands of high‐intensity sound generation; on the other hand, micro–nano technology should be used to manufacture miniature sound sources, which can more precisely control the generation of acoustic waves, thereby enhancing the intensity and directionality of the acoustic waves. In addition, digital signal processing technology should be employed to control and optimize the waveform and frequency of acoustic waves, aiming for higher intensity and better directionality. This may include adaptive filtering, beamforming technology, and phased array technology, as well as the development of intelligent systems capable of real‐time monitoring and adjustment of acoustic wave parameters to adapt to different application environments and requirements. Further, the coupling effects of acoustic waves with other physical fields (such as electromagnetic fields and thermal fields) can be studied to explore new mechanisms for enhancing acoustic waves; for example, combining electromagnetic fields to control the propagation path and intensity of acoustic waves. Finally, by integrating knowledge from multiple disciplines such as physics, engineering, and biology, interdisciplinary research can be conducted to explore the application potential of low‐frequency acoustic waves in various fields and to achieve new technological breakthroughs.

## 6 Conclusion

Low‐frequency acoustic waves hold extensive application prospects, encompassing medical treatments, underwater communications, and industrial applications. Due to their long wavelengths, strong penetration, and diffraction capabilities, low‐frequency sound waves can transmit over considerable distances. However, these inherent properties also pose significant challenges in achieving directional emission and energy enhancement. Currently, the manipulation of low‐frequency sound waves faces issues such as insufficient sound intensity, inadequate directional effects, and the bulkiness of manipulation equipment.

Methods for enhancing the intensity of low‐frequency acoustic waves, such as acoustic lenses, acoustic resonators, and plasma acoustic metamaterials, as well as directional control techniques such as acoustic parametric arrays, phased arrays, Mie resonators, and plasma acoustic metamaterials, are predominantly based on acoustic metamaterials. Although some of these technologies exhibit good performance in directional control and sound intensity enhancement and can achieve excellent manipulation of low‐frequency acoustic waves at deep subwavelength scales, they still face issues including narrow applicable frequency bands and limited application scenarios.

Addressing the limitations of current technologies, this article explores potential research directions and proposes improvement schemes from multiple perspectives, which include developing new materials, utilizing micro–nano technologies to design and fabricate acoustic structures, conducting multi‐physics field coupling studies, and developing intelligent control systems to improve acoustic wave control effects. Exceptionally, the article discusses how to use existing low‐frequency acoustic wave manipulation technologies to lower frequency bands and underwater applications, providing new ideas for future research. This review emphasizes the importance of directional technology research for low‐frequency acoustic waves, aiming to encourage more researchers to engage in this promising field of study.

## Conflict of Interest

The authors declare no conflict of interest.
